# Reply to Saleh, C. Comment on “Bažadona et al. The Interconnection between Carotid Intima–Media Thickness and Obesity: Anthropometric, Clinical and Biochemical Correlations. *Medicina* 2023, *59*, 1512”

**DOI:** 10.3390/medicina60071158

**Published:** 2024-07-18

**Authors:** Danira Bažadona, Martina Matovinović, Magdalena Krbot Skorić, Hrvoje Grbavac, Andrej Belančić, Branko Malojčić

**Affiliations:** 1 Department of Neurology, University Hospital Centre Zagreb, 10000 Zagreb, Croatia; 2 Croatian Referral Center for Obesity Treatment, Department of Endocrinology, University Hospital Centre Zagreb, 10000 Zagreb, Croatia; 3 University Psychiatric Hospital Vrapce, 10090 Zagreb, Croatia; 4 Department of Clinical Pharmacology, Clinical Hospital Centre Rijeka, 51000 Rijeka, Croatia; 5 Department of Basic and Clinical Pharmacology with Toxicology, Faculty of Medicine, University of Rijeka, 51000 Rijeka, Croatia

We would like to begin by expressing our gratitude for the interest shown in our research [1,2].

We agree that a composite carotid intima–media thickness (CIMT) measurement that includes the common carotid artery (CCA), the carotid bifurcation and internal carotid artery (ICA) could provide more accurate measurement, but this type of measurement was not conducted for several reasons. First, as already mentioned, there is no universally accepted standardized measurement protocol and most studies were performed using only CCA IMT [3]. CCA IMT is a well-known marker of atherosclerosis and its association with cardiovascular and cerebrovascular events has been proven repeatedly [4,5,6]. The measurement of CCA IMT is easier to obtain and is highly reliable with less variability in measurements [6,7].

To conclude, although we agree our study protocol has its limitations, we believe that it provided more feasible patient examination and more comparable results.
